# Metabolic regulation of the lysosomal cofactor bis(monoacylglycero)phosphate in mice

**DOI:** 10.1194/jlr.RA119000516

**Published:** 2020-04-29

**Authors:** Gernot F. Grabner, Nermeen Fawzy, Renate Schreiber, Lisa M. Pusch, Dominik Bulfon, Harald Koefeler, Thomas O. Eichmann, Achim Lass, Martina Schweiger, Gunther Marsche, Gabriele Schoiswohl, Ulrike Taschler, Robert Zimmermann

**Affiliations:** * Institute of Molecular Biosciences, University of Graz, Graz, Austria; †† Division of Pharmacology, Medical University of Graz, Graz, Austria; † Otto Loewi Research Center, and Center for Medical Research, Medical University of Graz, Graz, Austria; ** Center for Explorative Lipidomics, BioTechMed-Graz, Graz, Austria; § BioTechMed-Graz, Graz, Austria

**Keywords:** lipid metabolism, phospholipids, liver, pancreas, adipose tissue, nutritional state, insulin, body temperature, lysosome

## Abstract

Bis(monoacylglycero)phosphate (BMP), also known as lysobisphosphatidic acid, is a phospholipid that promotes lipid sorting in late endosomes/lysosomes by activating lipid hydrolases and lipid transfer proteins. Changes in the cellular BMP content therefore reflect an altered metabolic activity of the endolysosomal system. Surprisingly, little is known about the physiological regulation of BMP. In this study, we investigated the effects of nutritional and metabolic factors on BMP profiles of whole tissues and parenchymal and nonparenchymal cells. Tissue samples were obtained from fed, fasted, 2 h refed, and insulin-treated mice, as well as from mice housed at 5°C, 22°C, or 30°C. These tissues exhibited distinct BMP profiles that were regulated by the nutritional state in a tissue-specific manner. Insulin treatment was not sufficient to mimic refeeding-induced changes in tissue BMP levels, indicating that BMP metabolism is regulated by other hormonal or nutritional factors. Tissue fractionation experiments revealed that fasting drastically elevates BMP levels in hepatocytes and pancreatic cells. Furthermore, we observed that the BMP content in brown adipose tissue strongly depends on housing temperatures. In conclusion, our observations suggest that BMP concentrations adapt to the metabolic state in a tissue- and cell-type-specific manner in mice. Drastic changes observed in hepatocytes, pancreatic cells, and brown adipocytes suggest that BMP plays a role in the functional adaption to nutrient starvation and ambient temperature.

Lysosomes are the primary degradative organelles essential for the maintenance of metabolic homeostasis and health. Bis(monoacylglycero)phosphate (BMP), also known as lysobisphosphatidic acid, is a phospholipid highly enriched in the intraluminal vesicles (ILVs) of late endosomes/lysosomes (1) and plays a key role in lysosomal cargo sorting. BMP is a structural isomer of phosphatidylglycerol that harbors one FA at each of the two glycerol moieties. It has a unique *sn*-1-glycerophospho-s*n*-1′-glycerol stereoconformation, making it highly resistant to degradation by phospholipases of acidic organelles (2). Because it is negatively charged at a lysosomal pH, it can act as a docking platform that recruits positively charged lipid hydrolases to ILVs, thereby facilitating the degradation of lipid cargo (3). Additionally, BMP is an important cofactor in lysosomal cholesterol and sphingolipid metabolism through its interaction with cholesterol transport and sphingolipid activator proteins (4, 5). BMP is considered on the basis of these versatile functions to be a key activator of lipid sorting and digestion (6). Recent data also suggest that BMP is a cofactor for heat shock protein 70, a chaperone that promotes cell survival by inhibiting lysosomal membrane permeabilization (7).

Changes in the cellular BMP content reflect ILV formation and degradation in acidic organelles and therefore indicate an altered metabolic activity of endolysosomal compartments. Little is known about the physiological regulation of BMP. However, under pathophysiological conditions, BMP concentrations are increased in numerous lysosomal storage disorders (8) that can be caused by mutations in genes encoding lysosomal enzymes or as a side effect of drugs characterized by hydrophobic ring structures and side chains with a cationic amine group. These “cationic amphiphilic drugs” comprise many pharmacologic agents, including antibiotics, antidepressants, antiarrhythmics, cholesterol-lowering agents, and others (9). They accumulate in lysosomes and disturb lipid degradation, possibly by interacting with negatively charged BMP (6). Genetic as well as drug-induced lysosomal storage disorders are associated with increased BMP concentrations in tissues and in the circulation, which is utilized in clinical practice as a biomarker for the diagnosis of these disorders (10). We recently demonstrated that high-fat-induced metabolic disease is also associated with elevated hepatic and circulating BMP concentrations (11). Furthermore, published data demonstrate that excessive dietary lipids cause lysosomal lipid storage and BMP accumulation in the kidney (12). These changes possibly reflect adaptations to the dietary lipid overload causing an enhanced lipid flux through lysosomes, and on the basis of its versatile function in cargo sorting, it can be assumed that BMP is synthesized to facilitate metabolite flux through the disturbed lysosomal compartment. Accordingly, it was demonstrated that increased BMP levels induce cholesterol clearance in Niemann-Pick C disease in vitro and in vivo (13). Niemann-Pick C disease is characterized by the accumulation of unesterified cholesterol in late endosomal/lysosomal compartments (14) and is caused by mutations in the cholesterol transport proteins NPC1 or NPC2. Interestingly, increased BMP levels counteract cholesterol accumulation in cells lacking functional NPC1 but not NPC2, indicating that the interaction of BMP with NPC2 is essential for promoting cholesterol clearance (15).

Despite its importance in cellular physiology and its association with human disease, the molecular basics of BMP metabolism and its regulation remain elusive. To obtain fundamental insights into the physiological regulation of BMP, we studied the effects of nutritional and metabolic factors on BMP content and FA profiles. Drastic changes upon fasting and cold exposure implicate that BMP is important in the functional adaptation to changing metabolic conditions.

## MATERIALS AND METHODS

### Animals

Animal experiments were approved by the Austrian Federal Ministry for Science, Research, and Economy and the ethics committee of the University of Graz and conducted in compliance with the Council of Europe Convention. C57Bl6/J mice were bred and maintained on a regular light-dark cycle (14 h light, 10 h dark) at 22 ± 1°C in a specific pathogen-free barrier facility. A standard laboratory chow diet (R/M-H Extrudate; Ssniff Spezialdiäten GmbH, Soest, Germany) and drinking water were provided ad libitum unless otherwise indicated. Age-matched male mice were used for all studies.

### Isolation of hepatocytes and NPCs

Primary mouse hepatocytes and nonparenchymal cells (NPCs) were isolated as described previously with some modifications (16). In brief, mice were anesthetized, and the abdomen was surgically opened by a vertical incision. The liver was perfused via the portal vein with Krebs-Henseleit buffer (without Ca^2+^ and SO_4_^2−^) for 5 min, followed by a perfusion with Krebs-Henseleit buffer containing 0.2 mg/ml collagenase type CLS II (Worthington Biochemical Corporation, Lakewood, NJ), 2% BSA, and 0.1 mM CaCl_2_ for 10 min. Thereafter, the liver was excised and disrupted, and the cell suspension was passed through gauze and filtered through a 70 µm cell strainer. Hepatocytes were separated from NPCs by centrifugation at 50 *g* for 3 min at 4°C. The NPC fraction was pelleted by centrifugation at 900 *g* for 5 min at 4°C. Both cell fractions were washed twice with PBS before use.

### Separation of brown adipose tissue into adipocyte and stromavascular fractions

Fresh brown adipose tissue (BAT) depots were collected and freed from white adipose tissue (WAT). BAT was finely minced into small pieces and digested using collagenase type CLS II (1 mg/ml; 332 U/mg) and Dispase II (3.3 mg/ml; 0.9 U/mg; Sigma-Aldrich, St. Louis, MO) dissolved in PBS supplemented with 10 mM CaCl_2_ for 45–60 min at 37°C and 110 rpm. Enzymes were inactivated by the addition of DMEM containing 10% FBS. Cell suspensions were then filtered through a 100 µm cell strainer. To fractionate different cell types, cell suspensions were centrifuged for 10 min at RT and 200 *g*, and floating brown adipocytes were collected. To pellet cells from the stromavascular fraction, the remaining cell suspensions were centrifuged for 10 min at RT and 1,000 *g*. Supernatants were disposed, and the cells were treated with erythrocyte lysis buffer (154 mM NH_4_Cl, 10 mM KHCO_3_, 0.1 mM EDTA, pH 7.4) for 2 min and then washed with PBS. Fractionation of BAT from fed and fasted mice was performed in parallel.

### Isolation of pancreatic endocrine and exocrine cells

The pancreas was injected with 3 ml of 1.7 mg/ml Collagenase P (Sigma-Aldrich) in HBSS, removed, and incubated at 37°C for 20 min. To deactivate the collagenase, the pancreas was shortly vortexed with HBSS containing 10% FCS and then centrifuged three times at 200 *g* for 2 min. Afterward, the digested pancreas was passed through a 500 µm cell strainer and centrifuged at 200 *g* for 2 min. The pellet was resolved in HBSS, and endocrine and exocrine cells were separated by density gradient in Histopaque (Sigma-Aldrich). After centrifugation, endocrine and exocrine cells were collected and washed with HBSS three times. Exocrine cells were immediately stored at −80°C before use. Endocrine cells (pancreatic islets) were handpicked under a microscope and immediately stored at −80°C before BMP analyses.

### In vivo macrophage depletion

Mice were intravenously treated with 100 µl Clodrosome® (liposomal clodronate; Encapsula NanoSciences, Brentwood, TN) or Encapsome® (control liposomes; Encapsula NanoSciences) on day 1 and day 3, and tissues were excised on day 5. For relative gene expression analyses, liver and spleen RNA were used to prepare cDNA. To avoid DNA contaminations, 2 µg RNA were digested with 1 U/ml DNase I (Invitrogen, Carlsbad, CA) at 25°C for 15 min followed by heat inactivation of the enzyme at 65°C for 10 min. Thereafter, 1 µg RNA was transcribed using random primers and a High-Capacity cDNA Reverse Transcription Kit (Thermo Fisher Scientific, Waltham, MA). Eight to forty nanograms of cDNA were used for the PCR reaction using 10 pmol of forward and reverse primers, Maxima SYBR Green (Fermentas; Thermo Fisher Scientific), and the StepOnePlus Real-Time PCR System (Thermo Fisher Scientific). Relative gene expression was analyzed using the DD-Ct method (17) and normalized to 36B4. The following primers were used: F4/80 forward: 5′-GGATGTACAGATGGGGGATG-3′; F4/80 reverse: 5′-CATAAGCTGGGCAAGTGGTA-3′; 36B4 forward: 5′-GCTTCATTGTGGGAGCAGACA-3′; and 36B4 reverse: 5′-CATGGTGTTCTTGCCCATCAG-3′.

### Immunoblotting

Tissues were homogenized in buffer A (250 mM sucrose, 1 mM dithiothreitol, 1 mM EDTA, 20 µg/ml leupeptin, 2 µg/ml antipain, 1 µg/ml pepstatin, and Roche PhosphoSTOP) centrifuged at 1,000 *g* for 10 min, and 20 µg protein from the infranatant was subjected to SDS-PAGE, transferred to a PVDF membrane (Karl Roth GmbH, Karlsruhe, Germany), and blocked with 10% blotting-grade milk powder (Karl Roth GmbH) in TST (50 mM Tris/HCl, 0.15 M NaCl, 0.1% Tween-20, pH 7.4). Membranes were incubated with antibodies against phospho-AKT (Ser473; Cell Signaling Technology, Danvers, MA) (#9271), AKT (pan; Cell Signaling Technology) (#4691), GAPDH (#2118S) (Cell Signaling Technology), or UCP-1 (ab10983; Abcam, Cambridge, UK) prepared in 5% milk powder in TST. Antibody binding was detected with anti-rabbit HRP-linked secondary antibody (PI-1000; Vector Laboratories, Burlingame, CA) in 5% milk powder in TST and visualized using Clarity Western ECL Substrate and the ChemiDoc Touch Imaging System (Bio-Rad, Hercules, CA).

### Targeted BMP analysis

Total lipids of weighed tissue explants were extracted twice according to Folch et al. (18) using 4 ml chloroform-methanol (2:1; v/v) containing 500 pmol butylated hydroxytoluene, 1% acetic acid, and 150 pmol internal standard (14:0-14:0 BMP; Avanti Polar Lipids, Alabaster, AL) per sample. Extraction was performed under constant shaking for 90 min at RT. After the addition of 800 µl dH_2_O and further incubation for 30 min at RT, samples were centrifuged at 1,000 *g* for 15 min at RT to establish phase separation. The lower organic phase was collected, 2.5 ml chloroform was added to the remaining aqueous phase, and the second extraction was performed as described above (30 min at RT with subsequent centrifugation). Combined organic phases of the double extraction were dried under a stream of nitrogen and resolved in 200 µl methanol/2-propanol/water (6:3:1; v/v/v) for UPLC/MS analysis.

Chromatographic separation was modified according to Knittelfelder et al. (19) using an ACQUITY-UPLC system (Waters Corporation, Milford, MA) equipped with a Kinetex C18 column (2.1 × 50 mm, 1.7 µm; Phenomenex, Torrance, CA) starting a 15 min linear gradient with 100% solvent A [methanol-H_2_O (1/1; v/v), 10 mM ammonium acetate, 0.1% formic acid, and 8 µM phosphoric acid], allowing baseline separation of isomeric phosphatidylglycerol and BMP species.

An EVOQ Elite™ triple quadrupole mass spectrometer (Bruker, Billerica, MA) equipped with an ESI source was used for detection. Distinctive fragmentation was achieved for BMP (see below) and phosphatidylglycerol (respective diacylglycerol fragment) in the positive ionization mode. BMP species were analyzed by selected reaction monitoring using the generic mass transition [MNH4]^+^/[RCOO+58]^+^ (of the respective esterified FA) as the transition (23 eV collision energy, 150 ms dwell time, 0.7 resolution for Q1/Q3). Data were normalized for recovery, extraction, and ionization efficacy by calculating analyte/internal standard ratios; quantified via external calibration using BMP 36:2 (Avanti Polar Lipids); and expressed as mol/g tissue.

### Statistical analysis

Figures were prepared using GraphPad Prism 8 (GraphPad Software, San Diego, CA). Data sets are presented as means ± SDs. Statistical significance between two groups was determined by a Student’s unpaired *t*-test (two-tailed) or ANOVA followed by a Bonferroni or Dunnett’s post hoc test for multiple comparisons. *P* < 0.05 was considered statistically significant.

## RESULTS

### Tissue BMP content and FA composition are regulated by fasting and refeeding

**Fig. 1.**
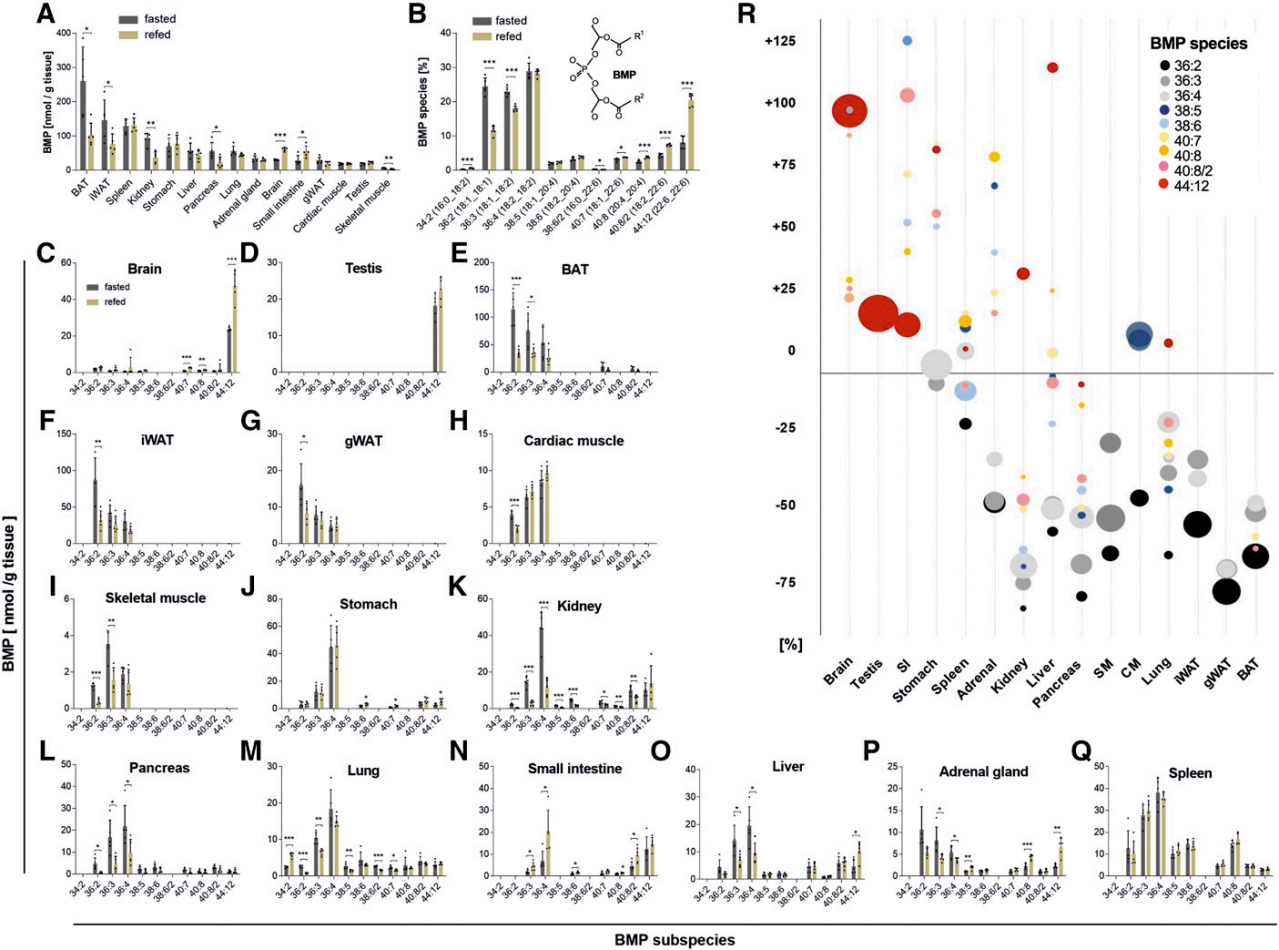
Tissue distribution and nutritional regulation of BMP content and FA composition. Tissue samples were obtained from mice fasted for 14 h (*n* = 5) or refed for 2 h after fasting (*n* = 5). A: The total BMP content of tissues was calculated as the sum of all BMP species shown in B. B: The relative abundance of molecular subspecies of BMP was calculated from all analyzed tissues shown in A. The structure of BMP 34:2 is shown in the insert. C–Q: BMP species distribution in tissues of fasted and refed mice. Lipid species are annotated as summarized carbon atoms:double bonds of the attached acyl chains. Data are presented as means ± SDs. Statistical significance was evaluated by an unpaired two-tailed Student’s *t*-test (*P < 0.05, **P < 0.01, and ***P < 0.001). R: Relative changes in BMP subspecies among all tissues upon refeeding. Bubble diameters indicate the relative mean quantity of BMP subspecies in the respective tissue.

To obtain basic insights into the nutritional regulation of BMP, we first determined BMP concentrations in tissues of fasted and 2 h refed mice. Tissues showed large variations in BMP content, with the highest abundance in BAT and lowest abundance in skeletal muscle in fasted mice (**Fig. 1A**). Refeeding decreased total BMP levels in BAT, inguinal WAT, the kidney, the pancreas, and skeletal muscle, whereas increased levels were observed in the brain and small intestine (Fig. 1A). The most abundant BMP species detected in mouse tissues are shown in Fig. 1B. BMP was predominantly esterified with oleic acid (present at 36:2, 36:3, 36:4, 40:7, and 38:5), linoleic acid (present at 36:3, 36:4, 38:6, and 40:8/2), and DHA (present at 40:7, 40:8/2, and 44:12). Refeeding caused a shift to a higher chain length and higher degree of unsaturation.

Notably, tissues strongly differed in BMP FA composition. The brain and testis almost exclusively contained BMP esterified with DHA (44:12) (Fig. 1C, D), while BAT, inguinal WAT, gonadal WAT, cardiac muscle, and skeletal muscle contained mostly BMP species esterified with linoleic and oleic acid (Fig. 1E–I). All other investigated tissues, including the stomach, liver, kidney, pancreas, lung, small intestine, adrenal gland, and spleen, contained 36:2/3/4 as well as species with higher chain lengths and degrees of unsaturation (Fig. 1J–Q). The relative tissue-specific changes in the BMP profile induced by the refeeding of fasted mice are summarized in Fig. 1R. Most notably, oleic and linoleic acid containing subspecies were decreased, whereas DHA containing subspecies were elevated in most tissues.

### Insulin treatment does not reproduce the refeeding-induced changes in tissue BMP levels

The observed effects of refeeding on tissue BMP profiles indicate that BMP metabolism is regulated by metabolic hormones secreted postprandially, such as insulin. Thus, we compared the effects of insulin treatment and refeeding in selected tissues. The effect of insulin was verified by the measurement of blood glucose concentrations, which were decreased by 30% 2 h after injection (93 ± 15 vs. 67 ± 5 mg glucose/dl; *P* < 0.05).

**Fig. 2.**
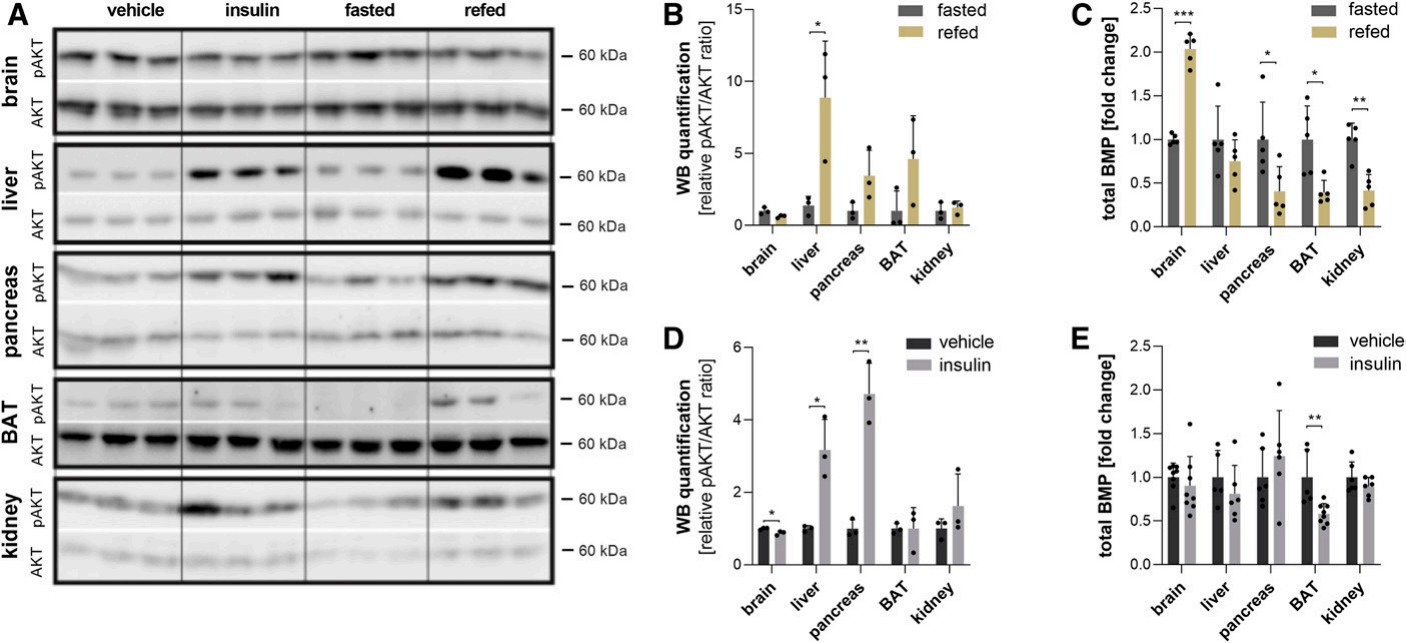
Effects of insulin on BMP content and FA composition. Mice were fasted overnight and given an intraperitoneal injection of vehicle (saline) or insulin (0.5 IU/kg), and tissues were collected after 2 h. A: Western blot analysis of phospho-AKT (pAKT, Ser437) and AKT (pan) as loading controls in tissue lysates and respective quantification upon (B) refeeding and (D) insulin treatment. C: Relative changes in total BMP content of tissues from refed mice (calculated from the data shown in Fig. 1) and (D) insulin-treated mice. Data are presented as means ± SDs. Statistical significance was evaluated by an unpaired two-tailed Student’s *t*-test (*P < 0.05, **P < 0.01, and ***P < 0.001; *n* = 5–8).

To compare insulin and refeeding effects in different tissues, we analyzed phosphorylation of the major downstream protein kinase AKT at serine 437, which is involved in the regulation of lysosomal biogenesis (20, 21) and therefore possibly also in the control of BMP metabolism. Refeeding enhanced AKT phosphorylation in the liver, pancreas, and BAT but not in the brain and kidney (**Fig. 2A**, B). Under these conditions, all tissues except the brain exhibited reduced BMP levels (Fig. 2C). Insulin treatment was associated with a significant increase in AKT phosphorylation in the liver and pancreas (Fig. 2A, D) but had only moderate effects on BMP levels in most tissues (Fig. 2E). Only BAT exhibited reduced BMP levels similar to the levels observed upon refeeding (Fig. 2E), but this was not associated with increased AKT phosphorylation (Fig. 2D). Thus, insulin treatment is not sufficient to reproduce refeeding-induced effects, and AKT activation does not correlate with changes in BMP levels. This suggests that BMP is regulated in a more complex manner, likely involving other hormonal and/or metabolic signals.

### Fasting increases BMP in hepatocytes but not in nonparenchymal cells

**Fig. 3.**
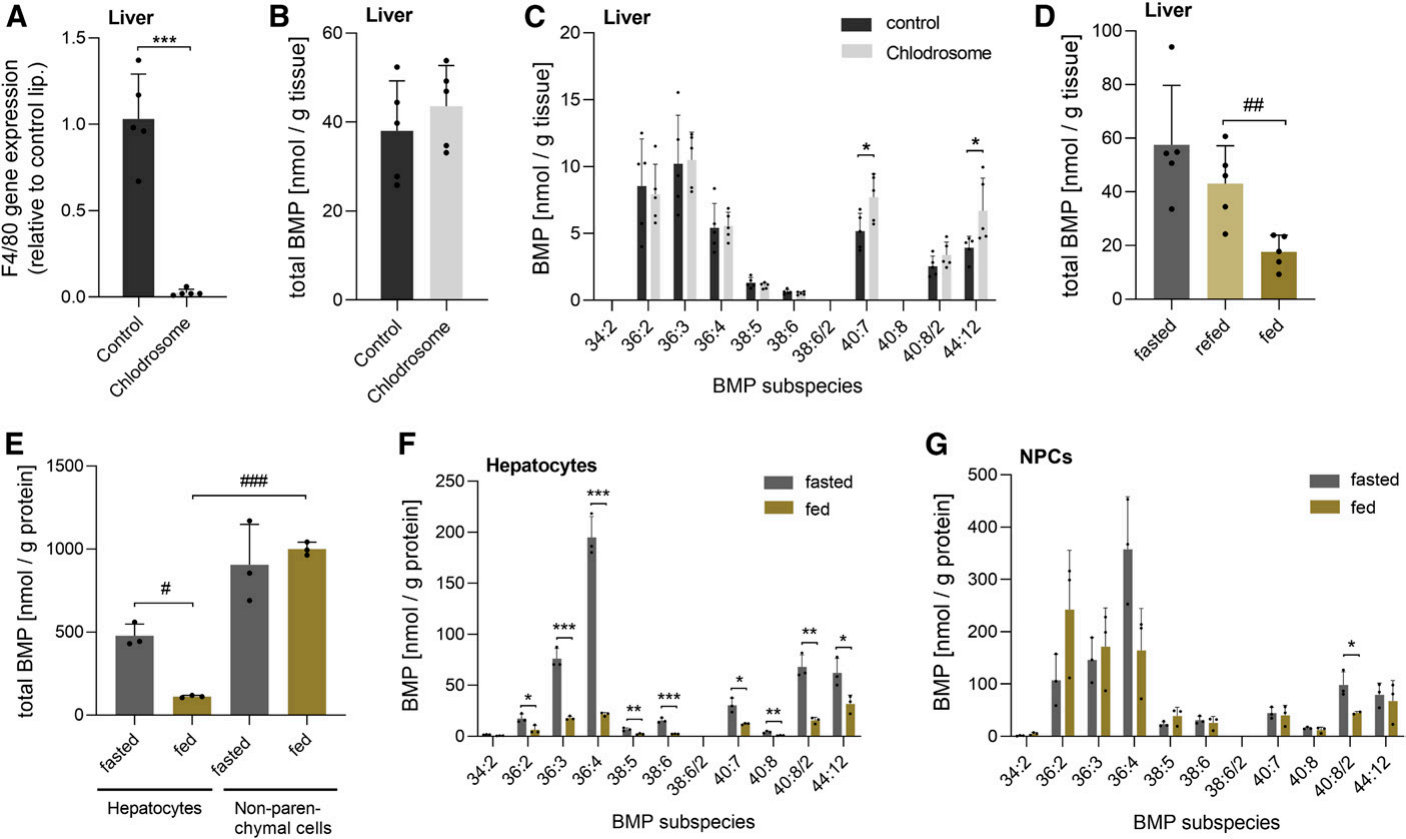
Distribution and nutritional regulation of BMP in the liver at the suborgan level. A: Expression of the macrophage marker F4/80 in mice treated with Clodrosome® (liposomal clodronate; *n* = 5) or empty liposomes as a control (*n* = 5). B: Total hepatic BMP content of Clodrosome-treated and control mice. C: BMP species distribution of Clodrosome-treated and control mice. D: Total hepatic BMP content of fasted, 2 h refed, and ad libitum fed mice (fed) (*n* = 5). E: Total BMP content of hepatocytes and NPCs isolated from fed and fasted mice (*n* = 3). F: BMP species distribution of hepatocytes. G: BMP species distribution of NPCs. Data are presented as means ± SDs. Statistical significance was evaluated by an unpaired two-tailed Student’s *t*-test (*P < 0.05, **P < 0.01, and ***P < 0.001) or ANOVA followed by a Bonferroni post hoc test for multiple comparisons (^#^*P* < 0.05, ^##^*P* < 0.01, and ^###^*P* < 0.001).

The tissue BMP profile reflects all resident cell types, and published data suggest that BMP is strongly enriched in macrophages (22), suggesting that tissue BMP concentrations could strongly reflect the macrophage content of tissues. To obtain insights into the suborgan distribution of BMP, we first investigated the contribution of macrophages to whole tissue BMP content in the liver, which possesses a relatively high abundance of resident macrophages (Kupffer cells). For this purpose, we treated mice with Clodrosome (liposomal clodronate), which selectively depletes macrophages. As indicated by the expression of the macrophage-specific marker F4/80, Clodrosome treatment almost completely depleted liver macrophages (**Fig. 3A**), but total hepatic BMP content remained unchanged compared with vehicle-treated controls (Fig. 3B). However, macrophage depletion caused a moderate shift to BMP with a higher FA chain length and unsaturation (Fig. 3C). These observations suggest that liver macrophages do not substantially affect whole tissue BMP content.

To further study the regulation of hepatic BMP levels, we compared the hepatic BMP content of fasted and refed mice to ad libitum fed mice (fed). Fed mice exhibited a reduction in BMP content compared with 2 h refed mice (Fig. 3D), suggesting that BMP degradation and remodeling is not completed after 2 h of refeeding. Next, we separated liver tissues from fasted and fed mice into parenchymal and NPCs. Notably, hepatocytes prepared from fed mice showed an 80% reduction in total BMP content compared with hepatocytes from fasted mice (Fig. 3E) due to a decrease in all detected BMP species (Fig. 3F). In contrast to hepatocytes, total BMP levels in NPCs were not affected by the nutritional state (Fig. 3E). However, NPCs from fed mice showed a moderate shift to a shorter FA chain length and higher degree of saturation (Fig. 3G). The BMP FA composition was similar in hepatocytes and NPCs, with the exception of 36:2 representing an NPC-enriched subspecies (Fig. 3F, G). In the fed state, NPCs exhibited 9-fold higher total BMP levels than hepatocytes (Fig. 3E). This suggests that NPCs substantially contribute to total hepatic BMP content. However, it must be considered that hepatocytes and NPCs constitute 80% and 6.5% of the liver volume, respectively (23). Based on the total BMP content in the hepatocyte and NPC fractions, we calculated that hepatocytes contain 79 ± 3% of total liver BMP in the fed state and 93 ± 2% in the fasted state.

### The nutritional state drastically affects BMP levels of pancreatic endocrine and exocrine cells

**Fig. 4.**
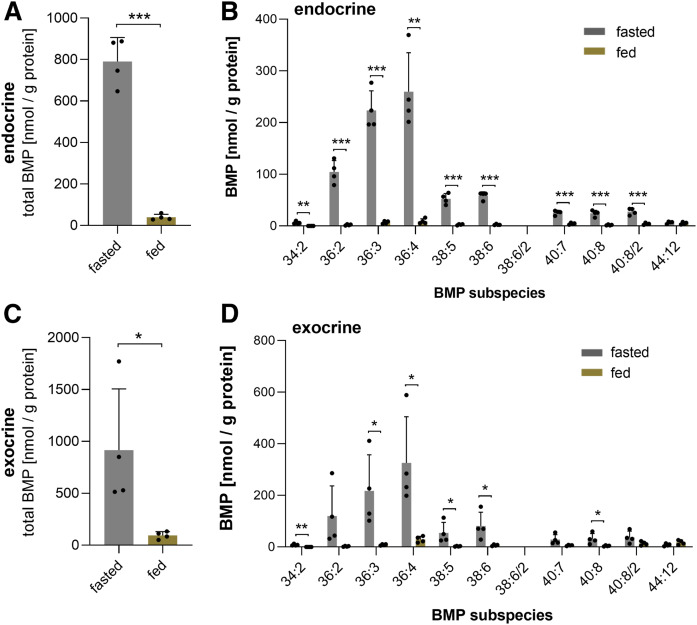
Nutritional regulation of BMP in the endocrine and exocrine pancreas. Total BMP content of endocrine and exocrine pancreatic cells (A, C) and respective BMP species distribution from fed and fasted mice (B, D) (*n* = 4). Data are presented as means ± SDs. Statistical significance was evaluated by an unpaired two-tailed Student’s *t*-test (*P < 0.05, **P < 0.01, and ***P < 0.001).

The central role of the pancreas in the regulation of energy homeostasis and the strong nutritional effect on pancreatic BMP levels (Fig. 1L) prompted us to investigate whether the nutritional state affects the BMP content of the endocrine and exocrine cells of the pancreas. As shown in **Fig. 4A**, we observed drastic differences in pancreatic islets isolated from fasted and fed mice. Compared with the fasted state, islets from fed mice exhibited a 96% decrease in total BMP content, with a reduction in all BMP species except 44:12 (Fig. 4B). We further analyzed total BMP content (Fig. 4C) and BMP subspecies (Fig. 4D) of exocrine cells and found a similar but less pronounced difference in fed and fasted mice. These results suggest that fasting strongly stimulates the formation of BMP-containing vesicles in endocrine and exocrine cells.

### Housing temperatures regulate the BMP levels of BAT

**Fig. 5.**
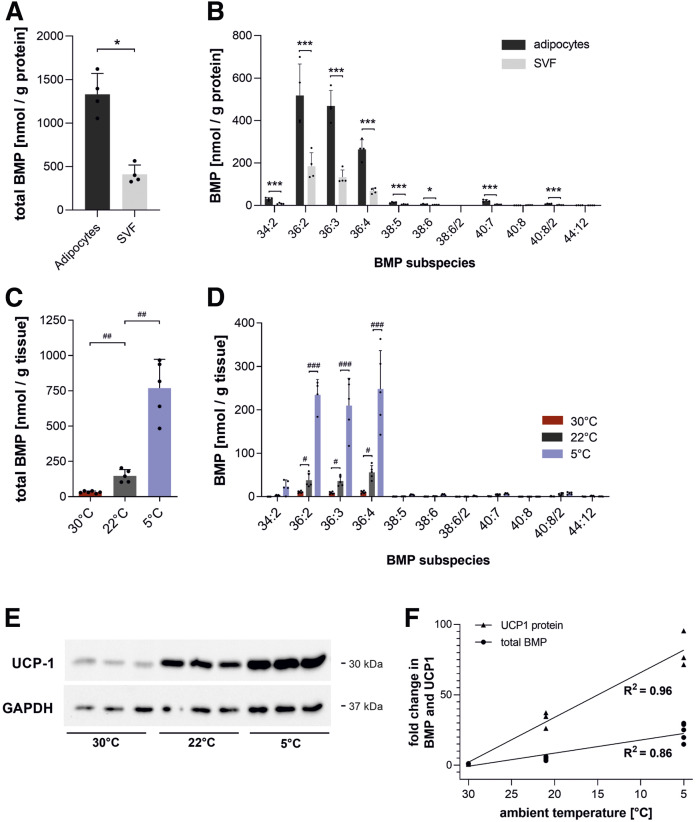
Effect of housing temperatures on BMP levels in BAT. A, B: Total BMP content and BMP species distribution in brown adipocytes and stromavascular cells (SVF) isolated from BAT of mice fed ad libitum at 22°C (*n* = 4). C, D: Total BMP content and BMP species distribution of BAT from mice housed at 22°C (*n* = 5), 5°C (*n* = 5), and 30°C (*n* = 6). E: Western blot analysis of UCP-1 protein in BAT lysates with quantification using GAPDH as a loading control. F: Correlation between total BMP content and UCP-1 expression in BAT at respective ambient temperatures. Data are presented as means ± SDs. Statistical significance was evaluated by an unpaired two-tailed Student’s *t*-test (*P < 0.05, **P < 0.01, and ***P < 0.001) or ANOVA followed by a Bonferroni post hoc test for multiple comparisons (^#^*P* < 0.05, ^##^*P* < 0.01, and ^###^*P* < 0.001).

BAT is crucial for thermal adaptation to ambient temperatures and among the tissues with the highest BMP content (Fig. 1A). To investigate the suborgan distribution of BMP in BAT, we separated BAT into adipocytes and stromavascular fraction. The BMP content in adipocytes was 3-fold higher than in the stromavascular fraction (**Fig. 5A**), suggesting that BMP is enriched in parenchymal cells. Both fractions contained BMP 36:2/3/4 as a major species (Fig. 5B). Upon cold exposure, BAT produces heat by nonshivering thermogenesis via UCP-1 and adapts its metabolic activity accordingly (24). To test whether BAT BMP content reflects its metabolic activity, mice were housed at standard conditions (22°C) or adapted to cold (5°C) and thermoneutrality (30°C) for 3 weeks. Remarkably, cold exposure increased BMP levels 5.3-fold, while thermoneutrality decreased BAT BMP content by 78% (Fig. 5C), predominantly due to changes in BMP 36:2/3/4 subspecies (Fig. 5D). As expected, UCP-1 expression was increased upon cold exposure (Fig. 5E), and both BAT BMP content and UCP-1 expression strongly correlated with housing temperatures (Fig. 5F).

## DISCUSSION

BMP facilitates the degradation and sorting of lipids in lysosomes delivered by endocytosis or autophagy and is therefore considered as an essential cofactor in maintaining metabolic homeostasis (25). Here, we provide novel insights into the tissue distribution of BMP and its physiological regulation. We observed that mouse tissues strongly differ in BMP content and show distinct BMP FA compositions. Furthermore, we demonstrate that fasting-induced lysosomal biogenesis and cold exposure affect BMP levels in a tissue- and cell-type-specific manner.

The comprehensive analysis of BMP revealed that tissues exhibit very characteristic BMP FA profiles that are similar to those described in a recent publication (26). The predominant BMP species in muscle and adipose tissue depots are esterified with oleic and linoleic acid. Other tissues, such as the liver and small intestine, comprise a broader spectrum of BMP species containing mono- and polyunsaturated FAs, whereas the brain and testis almost exclusively contain BMP esterified with DHA (44:12). BMP has been shown to activate many lysosomal lipid hydrolases and sorting proteins (6). However, most experiments have been performed with BMP esterified with oleic acid, and currently it is unknown whether DHA-containing BMP can also fulfill this function. It is well known that DHA is among the most abundant n-3 polyunsaturated FAs in the brain and testis, where it regulates important processes by affecting membrane structure or serving as a precursor of bioactive signaling lipids. An adequate supply of these tissues with DHA is required for brain health (27) and fertility (28). The high enrichment of DHA in BMP indicates that BMP affects DHA sorting in acidic organelles or acts as a short-term storage lipid for DHA. In accordance with this hypothesis, we observed that refeeding increases BMP 44:12 concentrations, indicating the incorporation of DHA when dietary sources are available.

The refeeding of fasted mice reduced the BMP content of several tissues, including BAT, kidney, and pancreas. In contrast, the small intestine and brain showed increased BMP levels in response to refeeding, demonstrating that BMP metabolism is regulated in a tissue-specific manner. Because the fasting response strongly varies among tissues (29), it is reasonable to assume that BMP, as an important lysosomal cofactor, similarly underlies tissue-specific regulation. The mechanism of BMP regulation is currently unclear. Thus, we investigated whether insulin, a major regulator of energy metabolism in the postprandial phase, is involved in this process. This seemed plausible because insulin is involved in the regulation of the mechanistic target of rapamycin complex 1 and transcription factor EB (TFEB), which controls lysosomal biogenesis (30, 31). Activation of the insulin receptor causes phosphorylation and activation of the major downstream effector AKT (protein kinase B) (30). AKT can directly phosphorylate and suppress TFEB (32), the master regulator of lysosomal biogenesis, and is also capable of activating the mechanistic target of rapamycin complex 1, which in turn prevents the nuclear translocation of TFEB (33). Accordingly, we investigated whether insulin treatment is sufficient to reproduce the refeeding-induced changes in BMP content. Insulin reduced total BMP levels in BAT comparable to refeeding but had no significant effect on other tissues. Furthermore, AKT phosphorylation did not correlate with changes in tissue BMP levels. This suggests that BMP metabolism is regulated in a more complex and tissue-specific manner, likely involving both hormonal and nutritional signals.

The tissue BMP profile reflects all resident tissue cell types. To get insights into the suborgan distribution of BMP, we separated parenchymal and NPCs in selected tissues. BMP was previously identified as a phospholipid enriched in macrophages that is linked to the high endosomal/lysosomal capacity of these cells (22). Nevertheless, the depletion of macrophages in the liver did not affect BMP content, suggesting a minor contribution of hepatic resident macrophages. We observed that NPCs exhibit a high BMP content, but total liver BMP content is predominantly determined by hepatocytes representing 80% of the liver volume. Notably, fasting induced a strong increase of BMP in hepatocytes but not in NPCs, indicating cell-type-specific regulation of BMP metabolism. This is likely due to the higher sensitivity of hepatocytes for the induction of starvation-induced autophagy, a lysosomal pathway that maintains cell function and survival through the degradation of cellular components (34).

The pancreas plays a key role in the regulation of energy homeostasis, and the strong nutritional regulation of BMP in the pancreas prompted us to investigate whether the nutritional state affects the BMP content of hormone-producing pancreatic islets and exocrine cells. Fasting was associated with a drastic increase of BMP in pancreatic islets and exocrine cells, indicating the formation of BMP-containing ILVs in acidic organelles. The functional aspects of BMP accumulation in pancreatic cells clearly require further investigations. Based on its important role in cargo sorting, it can be speculated that BMP-containing ILVs affect the storage, degradation, or release of hormones and digestive enzymes.

Remarkably, we found that BAT is among the tissues with the highest BMP content. Separating BAT into parenchymal cells and a stromavascular fraction confirmed that the majority of BMP is present in brown adipocytes, indicating a highly active lysosomal system. BAT is the major site for cold-induced nonshivering thermogenesis. It contains UCP-1 in the inner membrane of mitochondria, which dissipates the proton gradient from ATP production to generate heat (35). To sustain its function in thermogenesis, BAT relies on a continuous supply with energy primarily in the form of FAs (24) that can be delivered in two different ways. First, lipoprotein lipase exhibits a high local concentration and degrades triglycerides and releases FAs that are subsequently taken up via the FA transporter CD36 (36). Second, brown adipocytes can internalize whole lipoprotein particles via endocytosis (37). Importantly, the increased uptake and degradation of lipoproteins also requires an adaption of the endosomal/lysosomal system. We observed that both UCP-1 expression and BMP content strongly correlate with the housing temperature. Both parameters increase upon cold adaptation and decrease at thermoneutrality. Considering that BMP acts as activator of lysosomal lipid hydrolases (6), the increased BMP content may promote lysosomal degradation of lipoproteins and the subsequent liberation of FAs for mitochondrial oxidation and thermogenesis.

The observed changes in tissue BMP content in response to fasting or cold exposure depend on enzymes catalyzing de novo synthesis, degradation, and/or remodeling of BMP. We have recently shown that α/β hydrolase domain-containing 6 is involved in the degradation of BMP (11, 38). Concerning BMP synthesis, it is well established that phosphatidylglycerol is a precursor of BMP (8, 39). Yet the identity of enzymes catalyzing BMP synthesis starting from phosphatidylglycerol remains elusive. It is reasonable to predict on the basis of the strong differences in BMP FA composition between tissues that acyltransferases or transacylases catalyzing BMP synthesis in different tissues exhibit high selectivity for distinct FA species. Considering the important function of BMP in lysosomes, further elucidation of the metabolic pathways mediating BMP synthesis and degradation will provide important insights into the regulation of lysosomal function.

In conclusion, our observations demonstrate that tissues exhibit characteristic BMP profiles that adapt to the nutritional and metabolic state in a tissue- and cell-type-specific manner. The drastic changes observed in hepatocytes, brown adipocytes, and pancreatic cells suggest that BMP plays a role in the functional adaption to nutrient availability and ambient temperatures.

### Data availability
